# Macro-to-micro cortical vascular imaging underlies regional differences in ischemic brain

**DOI:** 10.1038/srep10051

**Published:** 2015-05-05

**Authors:** Suzan Dziennis, Jia Qin, Lei Shi, Ruikang K. Wang

**Affiliations:** 1 Department of Bioengineering, University of Washington, Seattle, Washington 98195, USA

## Abstract

The ability to non-invasively monitor and quantify hemodynamic responses down to the capillary level is important for improved diagnosis, treatment and management of neurovascular disorders, including stroke. We developed an integrated multi-functional imaging system, in which synchronized dual wavelength laser speckle contrast imaging (DWLS) was used as a guiding tool for optical microangiography (OMAG) to test whether detailed vascular responses to experimental stroke in male mice can be evaluated with wide range sensitivity from arteries and veins down to the capillary level. DWLS enabled rapid identification of cerebral blood flow (CBF), prediction of infarct area and hemoglobin oxygenation over the whole mouse brain and was used to guide the OMAG system to hone in on depth information regarding blood volume, blood flow velocity and direction, vascular architecture, vessel diameter and capillary density pertaining to defined regions of CBF in response to ischemia. OMAG-DWLS is a novel imaging platform technology to simultaneously evaluate multiple vascular responses to ischemic injury, which can be useful in improving our understanding of vascular responses under pathologic and physiological conditions, and ultimately facilitating clinical diagnosis, monitoring and therapeutic interventions of neurovascular diseases.

Stroke is caused by blockage or rupture of a blood vessel supplying the brain with oxygen and nutrients. The reduction of cerebral blood flow (CBF) below a critical level leads to a series of functional, and structural changes resulting in infarct^1^, while the peri-infarct region is potentially recoverable if treated promptly^2^. However, detailed vascular responses to ischemia still remain unclear. Over the past several decades, much effort has focused on neuroprotective mechanisms with little attention given to vasculature^2^. This is most likely due to the lack of a technology capable of elucidating real-time vascular dynamics *in vivo*. A more comprehensive understanding of both macro- and microvascular responses and hemodynamic parameters during and after ischemic injury would greatly improve our ability to develop therapeutic interventions aiming to improve vascular function and brain tissue survival after stroke.

Currently there are no imaging techniques for experimental stroke that can simultaneously and repeatedly measure blood volume, velocity, direction and tissue oxygenation, and monitor vascular remodeling (e.g. blood vessel deformation) down to capillary-level resolution on the same individual at an appropriate imaging depth without the use of contrast agents *in vivo*. Magnetic resonance imaging (MRI) measures CBF, volume and oxygenation that may or may not utilize contrast-enhancing paramagnetic agents^3^,^4^,^5^ and does not reliably detect irreversible damage in the first hours after stroke^6^. Positron emission tomography (PET) can determine the relationship between regional blood flow, volume and cerebral metabolic rate of oxygen^7^ but requires a radioactive tracer, making multiple assessments on the same individual potentially harmful. Both MRI and PET can access the greatest imaging depth (10 cm or more), but do not have the spatial resolution (~5 μm) required to visualize vessels at the capillary level. Confocal and multi-photon microscopy, are commonly used experimental techniques capable of ultrahigh resolution (~1 μm) imaging of CBF, but require the removal of intervening bone to access the cortex since the imaging depth is only limited to 200 μm^3^. These techniques also require injection of fluorescent tissue markers which may complicate the interpretation of the results.

To evaluate microvascular responses to experimental stroke, we developed a multimodal imaging system by combing optical microangiography (OMAG) with dual wavelength laser speckle (DWLS) imaging systems. OMAG is an optical-coherence-tomography (OCT)-based angiography technique that can provide 3D images of both tissue structure and blood flow at capillary level resolution with an imaging depth up to 2 mm without the need for exogenous contrast agents^8^,^9^,^10^,^11^. OMAG is capable of measuring cerebral blood flow non-invasively^9^,^12^, with equal accuracy as the gold standard that uses the radio-tracer of iodoantipyridine^13^. Through the use of Doppler principle, more recent improvements to OMAG, i.e., Doppler OMAG (DOMAG)^14^ allows for the detection of a wider range of fast and slow blood flow velocities for simultaneous measurement in capillaries, arterioles/venules and arteries/veins^15^. In our integrated system, we employed OMAG and DOMAG to map vascular responses down to the capillary level in a stroke model in mice.

Laser speckle contrast imaging (LSCI) is a popular tool used for the non-invasive study of vascular dynamics in experimental stroke^16^,^17^,^18^,^19^,^20^. DWLS imaging^21^ improves LSCI by adding a second laser wavelength into the system, allowing for the detection of the changes in oxygenated and deoxygenated hemoglobin concentration as well as the relative changes in CBF in the mouse brain, within tens of milliseconds, through an intact cranium^21^,^22^. Although DWLS does not provide useful depth information, it can provide a wide field of view imaging (~10 mm × 10 mm) and can be used to accurately predict the area of infarct within a minute after an occlusion^21^.

In this study, we used an integrated OMAG-DWLS to identify distinct blood flow regions corresponding to infarct, peri-infarct and mildly hypoperfused tissue, and to characterize the differences in vascular responses among these regions after middle cerebral artery occlusion (MCAO) in male mice. The multifunctional system detects microvascular responses with high spatial and temporal resolution before, during and after experimental stroke in the same animal over time. Here, we used DWLS to rapidly evaluate the CBF and hemoglobin oxygenation over the whole mouse brain at a macroscopic scale, upon which to guide the OMAG to hone into the desired regions to provide depth-resolved information regarding blood volume, blood flow velocity and direction, vascular architecture, vessel diameter and capillary density. The integrated OMAG-DWLS provides a yet unparalleled tool to assess key dynamic information about cortical cerebral blood flow, which is necessary to understand the mechanisms relating to vascular injury and repair.

## Materials and methods

### System configuration

**Figure 1:**
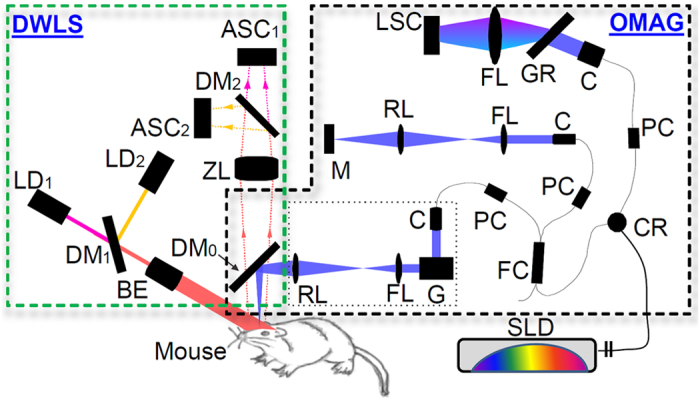
The schematic of the integrated OMAG-DWLS system, where ASC is the area scan camera, LSC the line scan camera, LD the laser diode (λ1 = 780 nm, λ2 = 825 nm), SLD the superluminescent diode, DM the dichroic mirror, ZL the zoom lens, BE the beam expander, RL the relay lens, FL the focus lens, C the collimator, G the galvo-scanner, GR the grating, PC the polarization controller, FC the fiber coupler and CR the optical circulator. [*Figure art by Ricky Wang].*

The schematic of the integrated system is shown in Fig. 1, which consists of DWLS (left) and OMAG (right) sub-systems. For the DWLS, two laser diodes, λ_1_ = 780 nm (LD1) and λ_2_ = 825 nm (LD2), were employed and combined coaxially by a dichroic mirror DM1. Both of the lasers were expanded and then uniformly illuminate the sample at an incident angle of ~60 ° from the tissue normal direction^21^. The illuminated area was ~10 mm in diameter that covers the whole mouse head under investigation. The back-scattered lights from the sample were transmitted to a zoom lens and then to two area-scan CMOS cameras (one for each wavelength) via another dichroic mirror, DM2. With the exposure time set at 10 ms, both cameras were configured to acquire images at a frame rate of 100 Hz with each frame consisting of 1000 × 1000 pixels, providing high contrast for speckle flow and blood oxygenation imaging. This imaging performance and spatiotemporal resolution are superior to previously reported methods in terms of frame rate and affordable pixel numbers^23^,^24^,^25^.

For the OMAG/OCT, a superluminescent diode (SLD) was employed that has a central wavelength of 1310 nm with a spectral bandwidth of 56 nm, giving a theoretical axial resolution of ~13 μm in air. A 2 × 2 optical coupler (FC) divided the light into two paths. The light in one path was transmitted towards a mirror in the reference arm, and the light in the other path was transmitted towards the mouse brain in the sample arm. In the sample arm, the light was coupled into an optical system which includes a collimator, a pair of galvo-scanners, and an objective lens with a 30 mm focal length, providing a lateral resolution of ~10 μm. The lights reflected from the sample and reference arms interfered and transmitted to a home-built high-speed spectrometer for the detection of the spectral interferogram with a maximum A-line rate of 92 kHz. The OCT signal was obtained through Fourier transformation of the detected interferogram following the algorithm described in^26^. The system sensitivity was determined to be ~105 dB with a light power on the sample at ~5 mW. To integrate DWLS and OMAG into one system, a dichroic beam splitter DM_0_ was used to bridge the lights from individual systems.

### Data processing

For the evaluation of brain hemodynamic responses, several parameters were calculated from the images captured by the integrated system. Firstly, the speckle contrast maps were calculated as the ratio of standard deviation to mean intensity, rendered by moving a 5×5 pixels binning window in the raw intensity image at λ1 = 780 nm. The relative flow changes were then derived based on the model of spatial speckle contrast versus flow velocity^16^. Secondly, the concentration changes in oxygenated hemoglobin (HbO) and deoxygenated hemoglobin (Hb) were evaluated by using the data acquired at λ1 (780 nm) and λ2 (825 nm), respectively, through a differential model based on the light absorption^19^. The change in total hemoglobin (HbT) was estimated by summing HbO and Hb changes.

The field of view of the OMAG was 2.0 × 2.0 mm^2^. 2 × 2 tiles of OMAG images were obtained to provide a large field of view area of 3.6 × 3.6 mm^2^ over the mouse brain. At each tile, 3-D volume data of both OMAG and DOMAG were acquired. The 3D OMAG data was composed of 512 pixels × 400 A-lines × 400 B-frames. Each B-frame was obtained from eight repeated frames with a frame rate of 180 Hz, using the OMAG algorithm described by Huang *et al.*^27^. The blood flow sensitivity of OMAG was ~4 μm/s, which is sufficient to resolve the complete vasculature morphology from arteries and veins down to the capillary level^10^. Subsequently, we used DOMAG for multi-range imaging of blood flow velocity. To extract the velocities ranged from arteries down to venules, we performed step scanning protocol with the A-line rate of 75 KHz as described in^15^. Twenty five repeated A-lines were captured at each position in one B-scan. Each B-frame contained such 380 positions, yielding a frame rate of 6 Hz. The 3D DOMAG data was composed of 512 pixels × 380 positions × 300 B-frames. Using this scanning protocol, the measured velocity range was scalable from [−2.0, 2.0] mm/s for slow flow measurement to [−18.2, 18.2] mm/s for fast flow measurement. More details can be found in^15^.

### Experimental procedure and animal preparation

All experiments were carried out in accordance with National Institutes of Health guidelines for research animal care and the protocols were approved by the Institutional Animal Care, and Use Committee at the University of Washington. Male mice C57BL/6 J (Charles River Laboratories, Hollister, CA) approximately 8–10 weeks of age with body weights from 20–25 g were used in all experiments.

Focal cerebral ischemia was induced in mice using the intraluminal MCAO model as described previously^28^. Briefly, the animal was anesthetized with 1.5% isoflurane (0.2 L/min O2, 0.8 L/min air). The body temperature was maintained at 36.5 ± 0.5 °C with a feedback rectal probe and heating pad (Harvard Apparatus, Holliston, MA). An incision of ~1.2 cm was made on the skin along the direction of sagittal suture, and the frontal parietal and interparietal bones were exposed. It is worth mentioning that the mouse cranium was left intact, neither thinned nor opened for imaging. The animal was then positioned under the OMAG-DWLS imaging system. After the baseline imaging, the animal was subjected to the MCAO procedure. During the procedure, a small laser Doppler probe (Model moorVMS-LDF2, Moor Instruments Ltd., Oxford, England) was affixed to the right side of the skull at the mid-ear to eye distance to monitor cortical perfusion and verify vascular occlusion and reperfusion. A 6-0 nylon filament with a silicone-coated tip was inserted into the right internal carotid artery via the external carotid artery (ECA) until the laser Doppler flowmetry value dropped to <25% of baseline. After 90 min of occlusion, the filament was removed, and reperfusion was allowed to occur. Subcutaneous buprenorphine (0.05 mg/kg) was administered every 8–12 h until the time of euthanasia. As part of the surgery, the ligation of the common carotid artery (CCA) was required to pass the filament through the ligated ECA to block the MCA. It is worth noting that the transient ligation of the CCA decreases blood flow to approximately 75% (data not shown) and was still in place during reperfusion, but not at the 24 h imaging time-point.

The integrated system (OMAG-DWLS) was utilized to assess important hemodynamic parameters, including the relative changes of blood flow, the changes in hemoglobin concentrations, blood flow direction and 3D blood vessel morphology. Briefly, we imaged animals prior to occlusion, at 90 min of occlusion and 20 min and 24 h after reperfusion unless otherwise indicated.

To quantify the infarct, 24 h after reperfusion brains were removed and stained whole with 1.2% triphenyl-tetrazolium chloride (TTC) in saline at 37 °C and photographed to overlay with the whole field images acquired with DWLS. Whole brains were then cut into 2 mm sections stained again with TTC and placed into 10% formalin overnight. Slices were photographed and analyzed for infarct size with SigmaScan Pro 5.0 software (Systat Software Inc., San Jose, CA, USA). TTC is metabolized by viable cells to a pink color, which can clearly delineate the area of infarct as the area of pallor^29^^,44,45^. Infarct volume was calculated by integrating infarcted areas across the rostral–caudal axis and expressed as a percentage of the contralateral hemisphere to account for edema.

### Statistical analysis

Differences in oxy-, deoxy- and total hemoglobin concentration, vessel diameter, cerebral blood volume and flow and capillary density were analyzed with one-way ANOVA for repeated measures with post hoc Newman–Keuls test. Hemoglobin concentrations were analyzed among infarct, peri-infarct, mild hypoperfusion and contralateral regions. Vessel diameter, cerebral blood volume and flow and capillary density were analyzed within each group. The criterion for statistical significance was p < 0.05. All values are reported as mean ± S.E.M.

## Results

In order to characterize the vascular responses to experimental stroke, male mice (n = 5) were subjected to occlusion of the MCA by the MCAO model^30^. Baseline images were taken immediately before the MCAO surgery. MCAO was confirmed by laser Doppler flowmetry. After 90 min of occlusion, the filament was removed from the imaging platform without moving the animal. After 24 h the animals were imaged, euthanized and brains removed and stained with TTC for the analysis of infarct size. The average infarct size was 46.65 ± 6.26% and 39.94 ± 4.75% in the cortex and total hemisphere, respectively. The survival rate for the surgical procedure was 75%.

**Figure 2:**
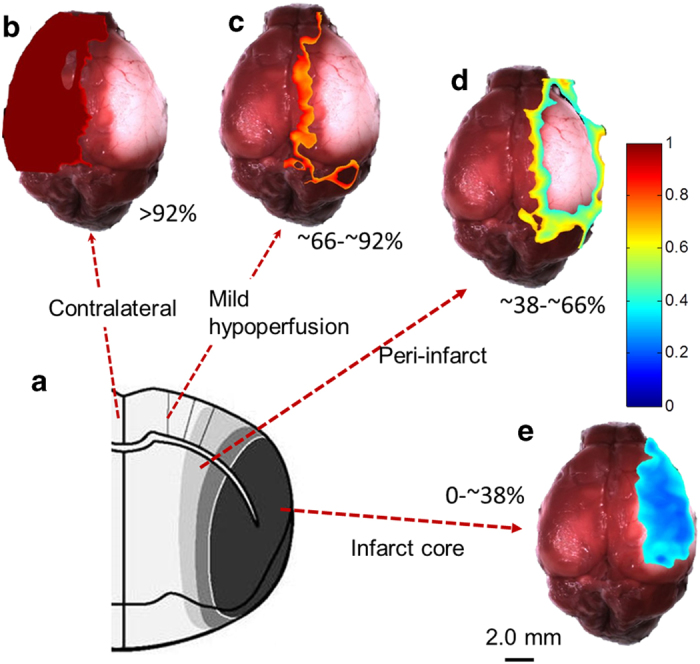
Differentiated infarct, peri-infact, mild hypo-perfusion and contralateral regions obtained from DWLS imaging results captured at 25 min after occlusion co-registered with TTC staining at 24 h of the mouse brain subjected to MCAO. (**a**) Anatomical depiction of four regions of differing blood flow rates: (**b**) contralateral (>92%), (**c**) mild hypo-perfusion (~66%–92%), (**d**) peri-infarct (~38%–66%) and (**e**) infarct core (<38%), respectively. Color bar represents the ratio of the ischemic flow relative to that of the baseline.

The reduction of blood flow below a critical value leads to irreversible neuronal death, resulting in infarction^31^. Reduced blood flow at an intermittent level in the peri-infarct zone leads to reversible damage^2^, and has been attributed to blood flow in the range of 40%–70%^32^. Fig. 2 shows four distinct blood flow regions according to the magnitude of blood flow reduction at 25 min of occlusion relative to pre-occlusion baseline values measured by DWLS. In the evaluated regions of five animals, the flow ranges were 92 ± 0.94%–100% for the healthy tissue region/contralateral (CT), 66 ± 2.77%–92 ± 0.94% for the region of mild hypoperfusion (MH), 38 ± 4.5%–66 ± 6.2% for the peri-infarct region (PI) and 0 – 38 ± 4.5% in the infarct core (IF). The infarct core flow map by DWLS consistently coregistered with the region of the infarct by TTC staining, similar to the findings reported in^33^.

**Figure 3:**
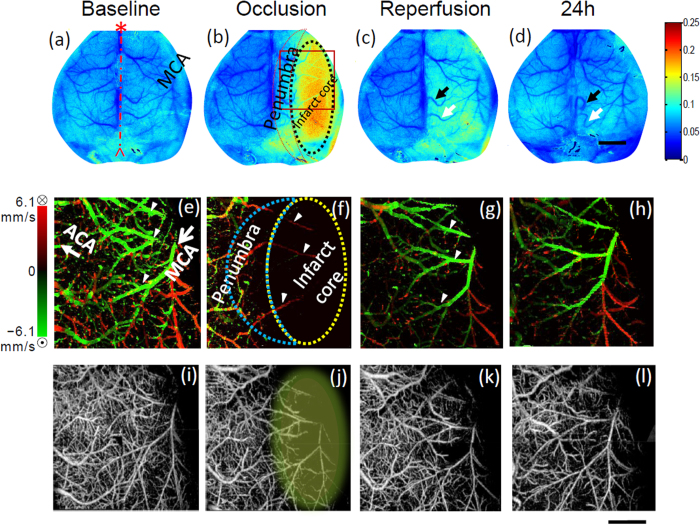
Representative DWLS maps (top row), velocity maps (middle row) and vessel morphology maps (bottom row) at four different time points: baseline, occlusion (25 min after the onset of MCAO), reperfusion (20 min after reperfusion) and 24 h after MCAO, respectively. The predicted infarct core is marked by black dotted region in (**b**) In (**a**) the dash-dotted line denotes the midline of mouse brain; the asterisk indicates the anterior of the brain. The MCA territory is denoted in (**a**). The region of interest for OMAG imaging is marked by the square in (**b**) Small arrows in (**e**) (**f**) and (**g**) label three branches of the MCA. The color bar for (**a-d**) stands for speckle contrast. Scale bar for DWLS maps = 2.0 mm. Scale bar for the DOMAG and OMAG images = 1.0 mm.

Regions and subtleties of dynamic CBF response before, during and after MCAO were revealed by the OMAG-DWLS system (Fig. 3). DWLS provided wide-field 2D mapping of overall blood perfusion within the brain. The infarct core, marked by the dotted region in Fig. 3b, was estimated from blood flow image based on the calculation of flow rate during occlusion normalized to the baseline flow rate (see Fig. 2). Prior to occlusion (baseline), blood flow in the right (ipsilateral) side of the brain was abundant with a highest value of 0, which is identical to that of the left (contralateral) brain (indicated by blue flow map). During occlusion, the blood flow decreased most prominently in the core region (blue dotted line Fig. 3b) of the MCA. A smaller peri-infarct region, surrounding the predicted core resulted in a less severe but marked decrease (area within the red line (Fig. 3b) and blue dotted line (Fig. 3f)). During reperfusion, the flow increased similarly over the entire hemisphere, with the infarct core and peri-infarct regions at similar levels. Vessel remodeling occurred in two surface meningeal vessels (marked by arrows in Fig. 3c at 20 min and Fig. 3d at 24 h). The predicted infarct area was validated by co-registering with TTC staining (data not shown).

A similar observation with a more detailed analysis was detected with high-resolution images of the microcirculation up to 2 mm in depth by OMAG. Quantification of blood flow requires the assessment of flow velocity in individual vessels; however, the flow velocity within the cerebral cortex ranges from tens of μm/s in capillaries to tens of mm/s in the MCA, a range that is too wide for the conventional Doppler OCT. To mitigate this problem, we used DOMAG to measure the flow velocity and direction in individual blood vessels. As expected, MCAO reduced blood flow drastically in the predicted infarct core. Flow direction in three large arterioles (marked by small arrows in (Fig. 3e,f,g)) that form anastomoses between ACA and MCA territories, reversed during occlusion. The flow in the arterioles returned to the baseline direction at the onset of reperfusion and was maintained at 24 h post-reperfusion. The images of vascular morphology are also demonstrated in Fig. 3I,l, from which we visualized the vascular architecture and its morphology changes in the blood vessels during occlusion and reperfusion in mouse brain. Prior to occlusion, the capillary network was intact and dense. During occlusion, both large vessel and capillary blood flow in the infarct core regions decreased dramatically, as shown by the lack of observed vessels. The large vessels recovered during reperfusion. Although capillary blood flow returned, it never fully recovered to baseline levels. After 24 h, the functional capillary flow (Fig. 3l) was also not fully recovered compared to that of the baseline (Fig. 3i).

**Figure 4:**
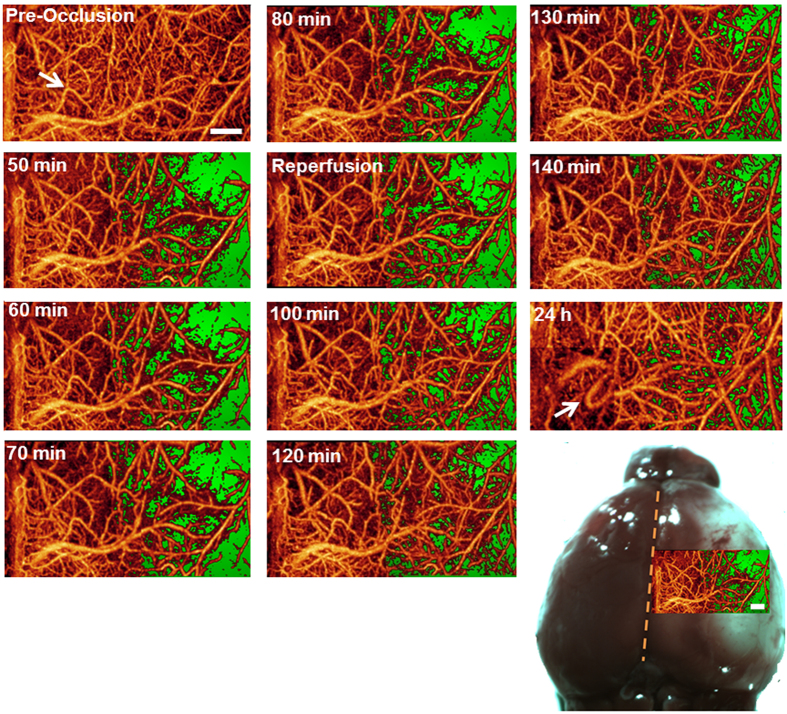
OMAG enables visualization of detailed blood flow responses during dynamic imaging and vascular remodeling after infarct has formed. Dynamic imaging of cortical blood flow using OMAG during MCAO (0 to 90 min) followed by reperfusion at 90 min. Consecutive OMAG images are shown at 10 min intervals. The green area depicts the lack of blood flow in the area of the MCA during occlusion. The white arrow highlights an area in which vascular remodeling occurred at 24 h after MCAO. Image size is 2.2 × 4.4 mm^2^. The image in the lower right is the OMAG image taken at 50 min overlaid on the 24 h infarct analysis by histological staining as the area of pallor. Scale bar = 500 μm.

Fast vascular responses (minutes) have not previously been visualized but the ability to do so will greatly aid in understanding the acute phases of vascular dysfunction and repair in ischemic injury. We developed a fast scanning approach for the OMAG system to allow for a rapid detection of blood flow response (Fig. 4). Our results show that in addition to the lack of complete reperfusion (green area) in the main vessels, reperfusion in many of the capillaries failed to occur suggesting that the lack of capillary reperfusion most likely contributes to the formation of the infarct. The capillaries did not re-perfuse to baseline levels, and vessel remodeling occurred at 24 h post-reperfusion.

**Figure 5:**
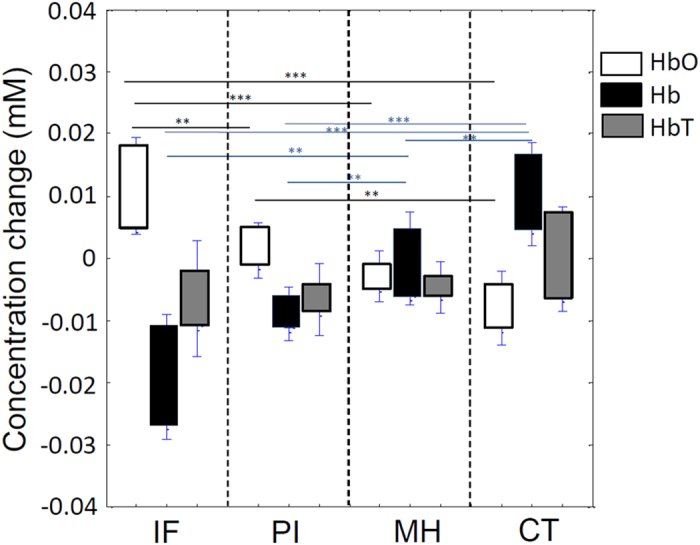
Concentration changes in oxygenated hemoglobin (HbO), deoxygenated hemoglobin (Hb), and total hemoglobin (HbT) from time of occlusion to reperfusion. *p < 0.05, **p < 0.01, ***p < 0.001.

The concentration changes of HbO, Hb and HbT in the predicted regions of infarct, peri-infarct, mild hypoperfusion and contralateral regions are shown in Fig. 5. The calculation was based on the comparison of HbO, Hb and HbT concentrations at reperfusion to that at occlusion. As expected, the change in hemoglobin oxygenation concentration from occlusion to reperfusion was highest in the infarct region because this region had the lowest level of HbO during occlusion. The averaged concentration change of HbO decreased as the distance of the defined region (PI, RF and CT) increased from the infarct core. Likewise, deoxygenated Hb showed an opposite and increasing averaged concentration at the time of reperfusion as the distance of the defined region (PI, RF and CT) increased from the infarct core. HbT was not significantly different among the four regions. In the mild hypoperfusion region, oxygenation status was relatively unaffected. An increase in Hb and decrease in HbO occurred in the contralateral region. Because the blood volume is linearly proportional to HbT^23^, HbT is an indicator of overall blood volume. However, upon reperfusion, the total hemoglobin remained slightly decreased in the IF, PI and hypoperfused regions. This phenomenon may be due to the vessel constriction during occlusion that did not recover during reperfusion, vessel constriction during reperfusion or vasodilatation during occlusion that recovered during reperfusion.

**Figure 6:**
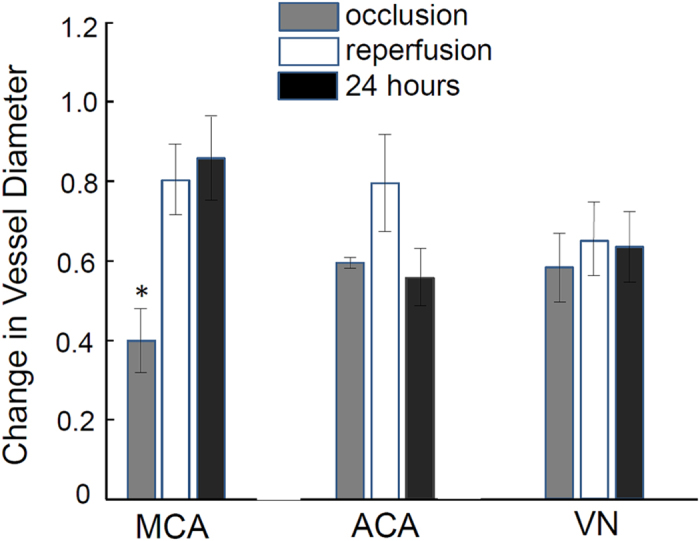
Vessel diameter change during occlusion, reperfusion and 24 hours after MCAO. Values in graphs are expressed as diameter change in cerebral vessels, including middle cerebral artery (MCA), anterior cerebral artery (ACA) and a direct branch of the superior sagittal sinus vein (VN), during time courses in the mouse brain subjected to the MCAO and presented as the means±S.E.M. (n = 5). *p < 0.05.

To determine whether vessel constriction or dilation occurred, OMAG was used to determine changes in vessel diameters (Fig. 6). The diameter of the MCA significantly decreased to ~40% during occlusion, recovered to ~80% of its original diameter during reperfusion and remained at a diameter similar to reperfusion, 24 h after reperfusion. The ACA vessel diameter decreased to ~58% during occlusion, although this decrease was not significant. The ACA vessel reperfused to ~79% and was decreased again to ~55% of baseline by 24 h. The vessel diameter in the vein was relatively constant at approximately 55%–62% during the time courses of occlusion, reperfusion and 24 hours.

**Figure 7:**
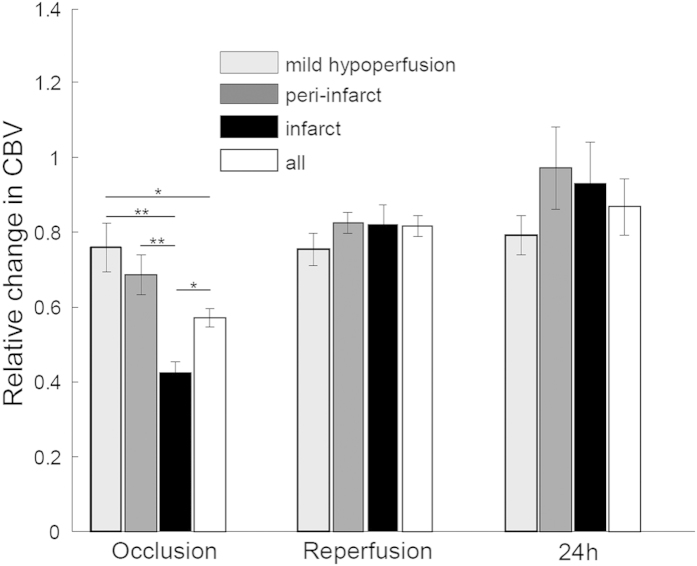
Cerebral blood volume responses in three major functional regions during occlusion and after reperfusion: infarct, peri-infarct and a mild hypoperfusion. All the data are normalized to baseline (n = 5). *p < 0.05, **p < 0.01.

Cerebral blood volume was calculated by thresholding the OMAG data (Fig. 7). At the time of occlusion, the regions from mild hypoperfusion to infarct suffered from a more pronounced reduction in blood volume. Due to occlusion of the MCA, the CBV reduction in the infarct region was severe; down to an average of 42% of baseline. Peri-infarct and mild hypoperfusion regions were less severe, with reductions to 68% and 76% of baseline, respectively. The standard errors of peri-infarct and mild hypoperfusion areas were larger than that of infarct area, indicating higher fluctuation in their vasculatures in response to occlusion. These data are consistent with those calculated by DWLS. During reperfusion, strong blood flow in the MCA led to the recovery of CBV in infarct and peri-infarct areas, about 82% and 83% of baseline, respectively. Changes in the MH region were minimal, possibly because it was with the farthest distance from the MCA. The regions of mild hypoperfusion, peri-infarct, infarct and the whole ipsilateral hemisphere (all) maintained similar reperfusion values, approximately 80% of baseline, illustrating the reperfusion effectiveness in the whole cerebral vasculature system. At 24 h after reperfusion, the blood volume in the peri-infarct was highest with 97% of baseline. The strong perfusion occurred in various types of vessels, including arteries, veins and capillaries. The blood volume in infarct area recovered to 93% of baseline, mainly due to the perfusion in MCA and its arterioles rather than the capillaries; the mild hypoperfusion area remained stable at 79%, just slightly higher than occlusion and reperfusion. The increased standard errors are likely due to the long-term compensatory variation among animals.

**Figure 8:**
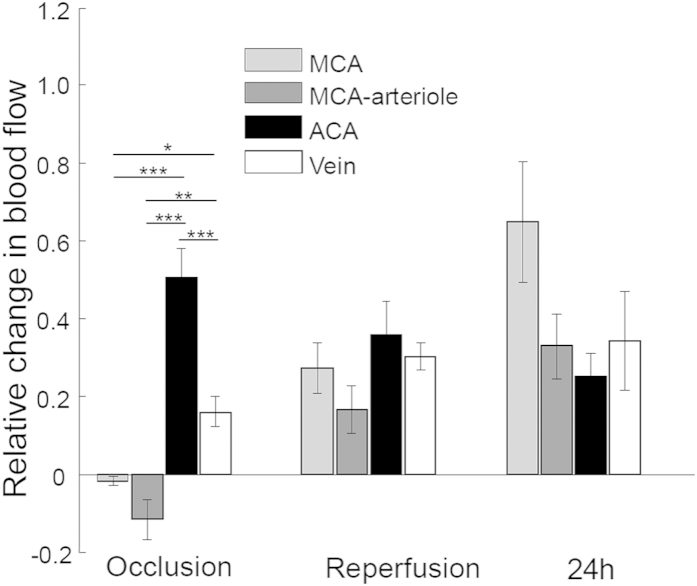
Cerebral blood flow responses in four main vessels during MCAO. The selected vessels are the MCA, the arteriole of the MCA, the ACA and a vein. Flow reversal in the MCA and its arteriole was observed during occlusion but returned to the original direction upon reperfusion. All data were normalized to baseline (n = 5). *p < 0.05, **p < 0.01, ***p < 0.001.

The blood flow in main vessels was calculated by integrating the axial velocity signals at a specific en face plane of each vessel from the DOMAG data as described in^34^. This calculation can determine the absolute blood flow without knowing *a priori* the Doppler angle between the vessel and probe-beam directions. As expected, occlusion reduced blood flow in the MCA almost to zero (Fig. 8). However, the remaining weak flow in the MCA, was −1.7% of baseline. The negative value indicates that the flow from the arteriole of MCA towards MCA reversed direction. This phenomenon may be due to compensation of blood flow from ACA via the anastomoses between the arterioles of the ACA and MCA. Flow reversal also occurred in the large arterioles of MCA, about −12% of baseline, which was stronger than in the MCA as they are closer to the ACA. The flow in the ACA during occlusion was also reduced, to about a half of the baseline. The ACA potentially served as the salvage artery to the areas originally supplied by the MCA. The blood flow to the vein was greatly reduced due to the overall reduction in the supply from arteries. After the onset of reperfusion, the MCA and its arteriole recovered to the normal flow direction, but the flow was slow compared to baseline. At the time point of 24 hours, the MCA and its arteriole recovered more, approximately 65% and 33% of baseline, respectively. The blood flow to the ACA was further reduced, to only 25% of baseline, and the blood flow to the vein was increased from the onset of occlusion to 24 h hours after reperfusion. Due to the long-term compensatory variation among animals, the standard error of blood flow in these main vessels, except ACA, is much higher at the 24 h time point.

**Figure 9:**
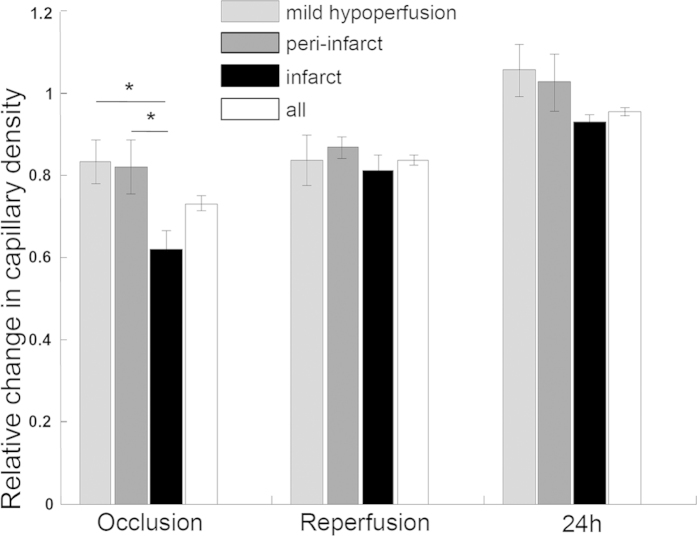
Capillary density responses in three major functional regions during MCAO: MH, PI and IF. For all the areas, the capillary density showed a similar distribution to CBV (Fig. 7). All the data are normalized to baseline (n = 5). *p < 0.05.

The response of capillary density most proximal to the skull (Fig. 9) showed a similar distribution to that of the CBV (Fig. 7) during MCAO. Capillary density increased from the time of occlusion to 24 hours after reperfusion. The capillary density responses were generally larger than the CBV responses, especially for the infarct area during occlusion and the hypoperfusion area at 24 hours after reperfusion. This difference indicated that the perfusion in the surface capillaries was better than in larger vessels.

## Discussions and Conclusion

We demonstrated an integrated imaging system, OMAG-DWLS that could be used to quantify detailed and longitudinal blood flow responses, vessel by vessel within four distinct and definable regions of CBF, upon ischemic injury. We used DWLS to rapidly assess the CBF and hemoglobin oxygenation over the whole mouse brain, macroscopically, upon which to identify the infarct, peri-infarct and mildly hypoperfused tissue based on relative cerebral blood flow changes. The information provided by DWLS was used to guide the OMAG to hone into the desired regions to provide depth-resolved information regarding blood volume, blood flow velocity and direction, vascular architecture, vessel diameter and capillary density. Such a comprehensive multi-parameter assessment of vascular responses has not previously been reported with other techniques. From OMAG, we obtained the following important findings: cerebral blood volume and blood flow progressively recovered from occlusion, reperfusion to 24 hours later. The flow direction in the MCA-arteriole(s) reversed direction during occlusion, and blood flow in both large vessels and surface capillaries was reinstated upon reperfusion.

We determined hemodynamic thresholds by DWLS starting from the final infarct. In the current model, the CBF below an average of 38% measured by DWLS can be consistently coregistered with the area of infarct. The very distinct region of moderate blood flow reduction was defined as the peri-infarct region. It appeared as the yellow and green regions in Fig. 3 and was easily distinguishable from both the mild hypoperfusion and infarct core regions in all animals. Although blood flow thresholds defining penumbra and infarct are variable due to differences in models and species, the blood flow threshold results reported here are consistent with other literature^2^,^6^,^18^ in that CBF less than 50% could be correlated with infarct after 24 h. Our data was presented as an average rather than a cut off threshold but the CBF from individual animals was always below 50%.

DWLS was able to predict the area of infarct within minutes after occlusion. Li Y *et al.*^18^ were also able to accurately predict infarct area with laser speckle imaging within the first minute after occlusion. They also used DWLS to determine occlusion of the MCA. Their system was set up so that the animal remained in the ventral position at all times. We used laser-Doppler flowmetry, rather than DWLS, to confirm MCAO in our model. Our system also has the capability to immediately assess the infarct region. However, because the OMAG-DWLS system requires imaging to be performed on the dorsal surface, after occlusion was confirmed with the animal in the ventral position, we were required to move the animal to a dorsal position to reposition the animal on the imaging platform. This movement may have caused a fluctuation in physiological parameters resulting in instablility of blood flow which we found to be stabilized after 20 min of occlusion (data not shown). In addition cortical spreading depolarizations may also cause fluctuations in blood flow after MCAO^35^. Therefore, we picked the time point at 25 min after inserting the filament to the middle cerebral artery as the time point to measure blood flow to correlate with infarct at 24 h.

We evaluated and measured blood flow velocity and direction, capillary architecture, hemoglobin oxygenation, vessel diameter and blood volume within four distinct and definable regions. Overall, the most prominent differences among the IF, PI MH and CT regions were observed during occlusion. The highest increase in HbO from occlusion to reperfusion occurred in the IF area where blood flow and oxygen are most severely compromised during ischemia. However, HbO also decreased in the CT region during reperfusion, suggesting that oxygenated blood was supplied by the contralateral side of the brain at this time. This may be due to the technical limitation that in addition to permanent ligation of the ECA, the CCA was also tied shut at this time. The CCA was untied after imaging when the reperfusion period was completed. We did not monitor blood pressure or blood gases largely due to that the amount of blood required to assess O_2_ and CO_2_ levels would greatly compromise the total hemoglobin concentration and survival of the animals. For this reason, we cannot rule out the possibility that systemic factors may have played a role in the changes of HbO and Hb data observed within each region. We assumed the optical path length is ~2.0 mm for the caculations of hemoglobin concentration changes using the two wavelengths^36^,^37^,^38^. Hemoglobin concentration values are approximate values.

MCAO decreased blood flow velocity and volume in the IF region more than any other region as expected and was accompanied by the highest decrease in vessel diameter (Fig. 6). These data are consistent with Shin *et al.*^39^ who reported vasoconstriction to be worse in areas where CBF is most severely compromised. Vasoconstriction was a result of neurovascular coupling in response to intense ischemic neuronal and astrocytic depolarization. Srinivasan *et al.*^40^ observed dilation in the MCA region and constriction in the ACA region. This study investigated this discrepancy and observed that vessel dilation and constriction may occur along a single artery depending on the earlier presence of nearby capillary non-perfusion. We measured the same area of vessel over time and cannot rule out that vasoconstriction was a result of either cortical spreading depression, proximal capillary perfusion or non-perfusion, or the lack of flow pressure in the artery itself. It is noticeable that the recovery of CBV in the MH was not as strong as in IF and PI areas (Fig. 7). One explanation for this observation is that the MH area had fewer large vessels and its main supply from the ACA was substantially reduced. As expected, the recovery in the peri-infarct area was larger than in the infarct area due to the successful rescuing compensation from ACA-territory during occlusion. CBV reached ~90% of baseline at 24 h after occlusion, suggesting a continued recovery of flow.

To determine the CBV, we used a fixed threshold to binarize the 3D OMAG data and then summarize all the values above the threshold to calculate blood volume. This threshold depends on the signal-to-noise ratio and the image quantifying scale. For our experimental conditions, we used a grayscale value of 50 as the threshold (~3dB above the noise floor). The corresponding angiography images clearly show various vessels from larger arteries down to capillaries [Figs. 3(i)-(l) and Fig. 4]. A higher or lower threshold would lead to smaller or larger values of blood volume at each time points, respectively. However, the relative changes between different time points would be very similar considering a not-so-large change in threshold. The 3D high-resolution imaging capacity of OMAG allowed us to resolve the blood flow signals down to unprecedented capillary level. However, the rapid changes in the phases of the optical signals in large vessels will lead to additional blood signal beneath these large vessels, showing as a shadowing artifact in the OMAG data. Thus, to minimize this effect, we employed a step down exponential filter to reduce this shadow artifact as described in^41^.

To obtain the CBF we integrated the DOMAG signals in a specific enface plane^34^. The binary threshold in the phase variance mask of DOMAG was used to determine the effective phase signals as described in^15^. Similar to the angiography threshold in OMAG, a higher or lower threshold would lead to smaller or larger blood flow value at each time points, respectively. However, the relative changes between time points would vary little. Due to the extended velocimetry range by DOMAG, we could accurately capture blood flow from fast arteries down to slow venules. Currently, the practical DOMAG technique cannot yield the velocity detection as slow as in capillaries, but there are some other emerging methods that may be employed to achieve this purpose^42^.

Srinivansan *et al.*^40^ found capillary perfusion disappeared and reappeared during occlusion and reperfusion respectively and observed a clearly defined region devoid of perfused capillaries, coregistered with the area of infarct. We also coregistered the area of infarct with distinct relative blood flow changes (DWLS) and lack of capillary perfusion (DOMAG) and were able to discern a clear border where the infarct was eventually formed. Although we measured main vessels rather than regions (Fig. 8), our results are in agreement with Srinivasan *et al.* in that blood flow reduction was most severe in the MCA with flow recovery not reaching baseline after reperfusion. Srinivasan *et al.* observed MCA flow to be greater than ACA flow after reperfusion. In contrast, we found flow was not higher in the MCA compared to the ACA after 1 h reperfusion, but was non-significantly higher at 24 h.

Capillary flow was drastically reduced in the infarct area and did not completely reperfuse during reperfusion and 24 h after MCAO (Figs. 3,4). However, when we quantified capillary density most proximal to the surface (Fig. 9), we found that it recovered to near baseline levels by 24 h after reperfusion. From the OMAG data, we quantified capillary density using the same threshold as for CBV to binarize the OMAG data. We removed the large vessels including arteries and venules by using their higher connectivity. The capillary density was finally obtained via dividing the area of the above-threshold data by the total area for each functional region as determined by DWLS. Due to the influence of the intact skull, the OMAG signal was detected more strongly near the skull, and we chose to calculate the density of the maximum enface projection view to avoid shadow artifacts. This may account for the differences observed with capillary flow in Fig. 3,4. Thus, capillaries most proximal to the skull may have reperfused to a greater degree than those of deeper within the cortex.

During occlusion, blood flow in three branches of the MCA (Fig. 3e,f) and an MCA-arteriole located in the IF region (Fig. 8) reversed direction. As the blood flow pressure of the MCA and its territories dropped during occlusion, the reversal of flow into the infarct region occurred through anastomoses between the ACA and the MCA. This phenomenon was accurately quantified in Fig. 8. Blood flow change remained the highest in ACA during occlusion compared to the MCA and vein. Upon reperfusion, blood flow continued to recover in the MCA, MCA-arteriole and the direct branch of sinus vein, but their recovery degrees were not similar. The highest increase in blood flow was observed in the MCA, followed by the ACA and lastly the direct branch of the sinus vein showing the smallest increase in blood flow. This may be due to increasing distances between the ACA and the sinus vein from the flow recovery in the MCA. The ACA is located in PI region and compensation between the ACA and MCA territory likely plays a key role in the survival of brain tissue. During reperfusion and at 24 h post reperfusion, the blood flow in the ACA decreased over time. This may be due to the interrelationship of blood flow among the global arterial system. Weaker flow in the ACA was accompanied by stronger flow in the MCA. When blood flow in the MCA recovered, it likely closed anastomotic connections between the ACA and the MCA. This is consistent with two-photon imaging^2^ which detected a reversal in the MCA territory and also suggested arteries in the MCA region were supplied by the ACA system during occlusion.

The above valuable hemodynamic information following experimental stroke was imaged and assessed with OMAG in combination with DWLS that determined functional CBF regions and served as the essential basis for OMAG quantifications. The integration of these two complimentary techniques paved the way to obtain the full set of hemodynamic and hemoglobin oxygenation information in the current study. To initially test the functionality of the novel OMAG-DWLS system we did not carry out long term time-points, however, OMAG-DWLS will be advantageous for the study of characterizing vascular dynamics of the penumbra and evolution of infarct in future studies. Clinically, this information will aid in the monitoring of infarct development and recovery. It may also contribute to other applications, such as locating abnormal vessels in tumors or monitoring vascular perfusion changes during progressive neurovascular disorders such as Alzheimer’s disease^43^.

In summary, the multifunctional OMAG-DWLS imaging system was useful in delineating microvascular hemodynamic and hemoglobin oxygenation responses to experimental stroke. The system is advantageous in the ability of localizing 2D microvasculature responses and in assessing depth-resolved individual vessel response. OMAG-DWLS can directly render images of any mouse model with local CBF and oxygenation variations. Overall, OMAG-DWLS may be useful to improve our understanding of vascular responses under pathologic and physiological conditions, and ultimately facilitate clinical diagnosis, monitoring and development of therapeutic interventions for neurovascular diseases^44^,^45^.

## Author Contributions

S.D. and J.Q. contributed equally to the work. R.W. conceived the project. S.D., J.Q., L.S. and R.W. designed research, performed experiments, analyzed data, and wrote the paper.

## Additional Information

**How to cite this article**: Dziennis, S. *et al*. Macro-to-micro cortical vascular imaging underlies regional differences in ischemic brain. *Sci. Rep.* **5**, 10051; doi: 10.1038/srep10051 (2015).
